# The Daily Work of the Heart

**Published:** 1881-05

**Authors:** 


### THE DAILY WORK OF THE HEART.

The following calculations are by Prof. Haughton.

1. Three ounces of blood are driven from each ventricle at each stroke of the heart.

2. The hydrostatical pressure on the left ventricle and aorta, against which the blood is forced out, amounts to a column of blood 9.923 feet in vertical height.

3. The muscular force of the left ventricle, in contracting, bears to that of the right ventricle the ratio of 13 to 5.

The daily work done by the left ventricle is 89.706 foot-tons, while that done by the right ventricle is 34.502 foot-tons, so that the total daily work of the heart is equivalent to a force lifting more thon 124 tons through one foot of vertical height.

